# Fasting plasma glucose variability and HbA1c are associated with peripheral artery disease risk in type 2 diabetes

**DOI:** 10.1186/s12933-019-0978-y

**Published:** 2020-01-07

**Authors:** Chun-Pai Yang, Cheng-Chieh Lin, Chia-Ing Li, Chiu-Shong Liu, Chih-Hsueh Lin, Kai-Lin Hwang, Shing-Yu Yang, Tsai-Chung Li

**Affiliations:** 1 Department of Neurology, Kuang Tien General Hospital, Taichung, Taiwan; 2 Department of Nutrition, Huang-Kuang University, Taichung, Taiwan; 3 School of Medicine, College of Medicine, China Medical University, Taichung, Taiwan; 4 Department of Medical Research, China Medical University Hospital, Taichung, Taiwan; 5 Department of Family Medicine, China Medical University Hospital, Taichung, Taiwan; 6 Department of Public Health, Chung Shan Medical University, Taichung, Taiwan; 7 Department of Public Health, College of Public Health, China Medical University, 91 Hsueh-Shih Road, Taichung, 40421 Taiwan; 8 Department of Healthcare Administration, College of Health Science, Asia University, Taichung, Taiwan

**Keywords:** HbA1c, Fasting plasma glucose, Glycemic variability, Peripheral artery disease

## Abstract

**Background:**

This study investigated whether visit-to-visit fasting plasma glucose (FPG) variability, as measured by the coefficient of variation (CV), increased peripheral artery disease (PAD) risk.

**Methods:**

Individuals with type 2 diabetes from the National Diabetes Care Management Program during the period 2002–2004, ≥ 30 years of age, and free of PAD (n = 30,932) were included and monitored until 2011. Cox proportional hazards regression models were implemented to analyze related determinants of PAD.

**Results:**

A total of 894 incident cases of PAD were identified during an average 8.2 years of follow-up, resulting in a crude incidence rate of 3.53 per 1000 person-years. Both FPG-CV and HbA1c were significantly associated with PAD after multivariate adjustment, with corresponding hazard ratios of 1.24 [95% confidence interval (CI) 1.04–1.47] for FPG-CV in the third tertile and 1.50 (95% CI 1.10–2.04) for HbA1c ≥ 10%. The findings of the sensitivity analysis remained consistent after excluding potential confounders, demonstrating the consistency of the results.

**Conclusions:**

The associations between HbA1c, variability in FPG-CV, and PAD suggest a linked pathophysiological mechanism, suggesting the crucial role of glycemic variability in clinical management and therapeutic goals in preventing PAD in type 2 diabetes.

## Background

Peripheral artery disease (PAD) remains a main cause of non-traumatic lower limb amputations and is linked with cardiovascular complications and long-term disability in type 2 diabetes [1, 2]. Early screening and interventions for modifiable risk factors for PAD can lead to reduction of risk and related complications. A number of traditional risk factors are linked with PAD [3, 4]; however, there is a need to further investigate non-conventional risk factors, especially in vulnerable populations.

PAD is defined as systemic atherosclerosis, mostly in the lower limbs [1, 3, 4]. Recent evidence suggested that glycemic variation might play a crucial role in the atherosclerosis pathogenesis and may confer additional risk for diabetes-related complications, independent of HbA1c level [5, 6]. In addition, there is increasing evidence suggesting that glycemic variability, as derived from the visit-to-visit fasting plasma glucose (FPG) measurements by coefficient of variation (CV), is an independent risk factor for ischemic stroke, diabetic peripheral polyneuropathy, Alzheimer’s disease, and all-cause or cause-specific mortality in persons with type 2 diabetes [7–11]. Moreover, animal and in vitro studies have also shown that oscillating glucose levels may have more harmful effects, including increased atherosclerosis, than chronic sustained hyperglycemia, that are involved in the pathogenesis of PAD [12–14]. However, no large-scale studies to date have specifically evaluated the relationship between PAD and variability in glycemic level, as measured by FPG-CV. Therefore, this large, retrospective cohort study investigated whether variability in glycemic level, as estimated by FPG-CV, has independent and significant associations with PAD in persons with type 2 diabetes.

## Methods

### Study population

Our data were extracted from 2 nationwide, population-based databases. The National Diabetes Care Management Program (NDCMP) under the Taiwan National Health Insurance (NHI) system was used to obtain diabetes outcome data for 2002–2004. In addition, the NHI administrative database was used for both baseline characteristics and follow-up assessments during the entry (or index) date and December 31, 2011. Additional financial incentives provided by The NDCMP for high-quality healthcare services such as increased frequency of monitoring, annual diabetes-related physical examinations and laboratory tests, and enhanced self-care education [15]. The program requires health care providers in the specialties of nephrology, family medicine, endocrinology, cardiology, internal medicine, and others to participate in clinical training and education programs for certification in Taiwan’s Diabetes Shared Care system program. These providers become eligible for participation of this program and voluntarily enroll patients with diabetes to this program. The purpose of these continuing clinical training and education programs is for enhancing the quality of care through clinical practice standardization, including assessment and diagnosis of diabetes complications. The coordinated physician-led multidisciplinary teams, including physicians and medical care staff, are responsible for the care of patients with diabetes as adhering to established clinical guideline. A physical or history examination suggestive of PAD required confirmed diagnosis by the ankle-brachial index (ABI) [16]. The physician-led multidisciplinary teams can refer patients requiring further diagnostic evaluation to a specialist.

A national retrospective cohort study, the Taiwan Diabetes Study, included 63,084 Chinese individuals with diabetes who participated in the NDCMP during 2002–2004. The index date was the NDCMP entry date. Individuals with a clinical diagnosis of diabetes according to the American Diabetes Association (ADA) criteria [International Classification of Diseases, ninth revision, Clinical Modification (ICD-9-CM) diagnosis code 250] were invited by their physicians to enroll. The NDCMP initially included 63,084 diabetic patients during 2002–2004. Of these, we excluded persons with PAD (ICD-9:443.9 or 440.21), type 1 diabetes (ICD-9: 250.x1/x3), or gestational diabetes (ICD-9: 648.83), and those aged < 30 years at baseline (Additional file 1: Figure S1).

Enrollees underwent comprehensive health assessment at the time of NDCMP entry to assess systolic blood pressure (SBP) and diastolic blood pressure (DBP), body measurements, blood and urine tests, and history of diseases and complications were also obtained. A standardized, computerized questionnaire was designed to collect information from all participants, which was administered by a case manager to record medication use, previous or current disease status, and lifestyle habits. Blood was drawn from an antecubital vein in the morning after a 12-h overnight fast and was sent within 4 h for analysis of FPG, HbA1c, low-density lipoprotein-cholesterol (LDL-C), triglyceride (TG), high-density lipoprotein-cholesterol (HDL-C), and creatinine. The interval for follow-up was every 3–6 months. All patients repeated the tests on each follow-up anniversary as those performed during program entry.

Participants with missing data for lifestyle behaviors, sociodemographic and diabetes-related factors, diabetic micro- and macrovascular complications, blood biochemical indices, and comorbidities were excluded. Those lost to follow-up and those with less than 3 months of follow-up including those with an entry date until PAD, death, or withdrawal from the NHI of less than 3 months were also excluded from the analysis. The last criterion was used to rule out the potential bias due to reverse causality. Finally, 30,932 patients were included in the study. The study protocol had been approved by the Ethical Review Board at China Medical University Hospital (CMUH102-REC3-016). Informed consent of the study participants was not required because the dataset used in this study consists of de-identified secondary data released for research purposes.

### Data sources for assessments at baseline and follow-up visits

The Taiwan government launched the NHI program in 1995, which covered nearly 99% of the entire 23.74 million Taiwan population in 1999 [17]. In 2014, more than 99.6% of the population was covered by the NHI program while 100% of hospitals and 92% of clinics island-wide were under the contracts with the NHI Bureau. The NHI Research Database consisted of information on demographics, prescriptions, and diagnoses from inpatient and ambulatory care claims. The proportion of enrollee withdrawals was very low because of the comprehensive coverage of the NHI program, and bias due to loss of follow-up was thus negligible. To enhance the validity of the claims data, the NHI Bureau in Taiwan sampled the medical charts on a routine basis every 3 months and anonymous experts randomly reviewed 50–100 inpatient and ambulatory care claims from each hospital and clinic, for improvement of the accuracy of coding [18]. The NDCMP was also covered by the NHI program. This study used the datasets for inpatient care based on admission and ambulatory care visits from 2002 to 2011. Every individual had a unique personal identification number (PIN). For privacy and security purposes, patient identity data in the NHI Research Database were cryptographically scrambled. Each patient was linked with the PIN in all NHI datasets without being identified.

### Ascertainment of outcome

The primary outcome was PAD, identified through record linkage with inpatient care and ambulatory care data in the NHI Research Database. The PAD incidence was based on coding according to the ICD-9-CM as 443.9 or 440.21. To enhance the accuracy of PAD diagnoses by excluding false positives, all PAD incidences met at least 1 of the following criteria: at least 1 inpatient or 3 ambulatory care claims. The study cohort was then followed up from the entry (index) date to December 31, 2011, or until a PAD event, withdrawal from the NHI, or death. A total of 894 newly diagnosed PAD patients were identified with an average of 8.20 years of follow-up.

For the 1-year period preceding cohort entry, ambulatory care and inpatient claims data were used to determine comorbidities, based on at least 3 ambulatory care claims or 1 claim for inpatient admission, including coronary artery disease.

Comorbidities within 12 months prior to the index date were tabulated using outpatient and inpatient claims data, based on at least 3 ambulatory care claims or 1 inpatient care claim. Any history of hypoglycemia (ICD-9-CM codes 250, 251.0–251.2), hyperlipidemia (ICD-9-CM code 272), hypertension (ICD-9-CM codes 401–405), atrial fibrillation (ICD-9-CM code 427.31), coronary artery disease (CAD) (ICD-9-CM codes 410–413, 414.01–414.05, 414.8, and 414.9), congestive heart failure (CHF) (ICD-9-CM codes 428, 398.91, and 402.x1), chronic hepatitis (ICD-9-CM codes 571, 572.2, 572.3, 572.8, 573.1, 573.2, 573.3, 573.8, and 573.9), cancer (ICD-9-CM codes 140–149, 150–159, 160–165, 170–175, 179–189, 190–199, 200, 202, 203, 210–213, 215–229, 235–239, 654.1, 654.10, 654.11, 654.12, 654.13, and 654.14), albuminuria (ICD-9-CM code 791.0), retinopathy (ICD-9-CM codes 250.5 and 362.0), or chronic obstructive pulmonary disease (COPD) (ICD-9-CM codes 490–496) was recognized as a comorbidity.

### Statistical analysis

The FPG-CV measurements for each patient from ambulatory visits within the 1st year of the entry (index) date were calculated for those with more than 2 FPG results. The average number of FPG measurements for this sample was 3.07, with a standard deviation of 0.95. The FPG-CV was divided by the square root of the ratio of total visits, divided by total visits minus 1 to correct for impact of the number of visits might on variation [19]. The multiple imputation approach was applied to handle missing CVs [20, 21]. Patients were grouped into tertiles according to FPG-CV. The extended Cox proportional hazards model with the Lunn-McNeil method was employed to weigh competing risks of PAD, and all-cause mortality was assessed by fitting a proportional sub-distribution hazards regression model focusing on cause-specific hazards for a competing risk of death [22]. Age, sex, and traditional variables were adjusted to compute hazard ratios (HRs) and their corresponding 95% confidence intervals (CIs). FPG-CV and HbA1c were assessed simultaneously with 3 multivariate models, with covariates being entered in hierarchical order: (1) adjustment for age (continuous) and sex; (2) additional adjustment for alcohol consumption (yes/no), smoking and tobacco use (yes/no), diabetes duration, antihypertensive treatment (yes/no), type of hypoglycemic drugs (no medication, 1 oral hypoglycemic drug, combination of 2, 3, or > 3 oral hypoglycemic drugs, insulin monotherapy, and insulin plus oral hypoglycemic drugs), and obesity defined as body mass index ≥ 27 kg/m^2^, the obesity criteria of the Ministry of Health and Welfare in Taiwan; and (3) additional adjustment for HbA1c (< 6, 6–8, 8–10, ≥ 10%), congestive heart failure, coronary artery disease, hypoglycemia, atrial fibrillation, hypertension, cancer, chronic hepatitis, chronic obstructive pulmonary disease, and hyperlipidemia. In addition, restricted cubic splines in Cox models were used to evaluate whether a linear or non-linear relationship of FPG-CV and HbA1c existed as a continuous variable with the risk of PAD. For sensitivity analyses, patients with diabetic ketoacidosis, hyperglycemic, hyperosmolar nonketotic coma, atrial fibrillation, myocardial infarction, and hypoglycemia were excluded (n = 1728). The statistical significance was defined as a 2-tailed p < 0.05. The SAS statistical package for Windows (Version 9.4, SAS; Cary, NC, USA) was used for analyses.

## Results

There were 894 incident cases of PAD after an average 8.2 years of follow-up with a crude incidence of 3.53 per 1000 person-years (3.63 for men, 3.44 for women). The incidence of PAD was 2.93, 3.61, and 4.06 per 1000 person-years in the first, second, and third tertiles of FPG-CV, respectively. The Pearson correlation coefficient between HbA1c and FPG-CV was 0.235, showing a positive weak relationship. The baseline characteristics grouped according to PAD status was presented in Table 1. There were differences according to diabetes duration, type of hypoglycemic medication use, hypertension medication, CAD, and retinopathy.

**Table 1:** Baseline characteristics according to peripheral vascular disease status in persons with type 2 diabetes enrolled in the National Diabetes Care Management Program, Taiwan (n = 30,932)

Variables	Peripheral vascular disease		p value
	No (N = 30,038)	Yes (N = 894)	
Socio-demographic factors			
Male, n (%)	14,008 (46.63)	421 (47.09)	0.81
Age (years), mean (SD)	60.99 (11.16)	61.73 (10.87)	0.05
Lifestyle behaviors, n (%)			
Smoking	4517 (15.04)	143 (16.00)	0.46
Alcohol drinking	2518 (8.38)	67 (7.49)	0.38
Diabetes-related variables			
Duration of diabetes (years), mean (SD)	6.80 (6.75)	7.97 (7.41)	< 0.001
Type of hypoglycemic drug use, n (%)			0.006
No medication	365 (1.22)	5 (0.56)	
One oral hypoglycemic drug	5134 (17.09)	113 (12.64)	
Two oral hypoglycemic drugs	12,536 (41.73)	353 (39.49)	
Three oral hypoglycemic drugs	5537 (18.43)	175 (19.57)	
> 3 oral hypoglycemic drugs	1575 (5.24)	59 (6.60)	
Insulin	885 (2.95)	18 (2.01)	
Insulin+ oral hypoglycemic drug	4006 (13.34)	171 (19.13)	
Drug-related variables, n (%)			
Hypertension drug treatment	11,671 (38.85)	316 (35.35)	0.04
Comorbidity, n (%)			
Obesity (BMI ≥ 27)	10,927 (36.38)	304 (34.00)	0.16
CAD	2603 (8.67)	99 (11.07)	0.01
Stroke	1519 (5.06)	52 (5.82)	0.35
CHF	785 (2.61)	19 (2.13)	0.43
Cancer	630 (2.10)	19 (2.13)	1.00
Hyperlipidemia	7621 (25.37)	220 (24.61)	0.63
Hypertension	13,736 (45.73)	397 (44.41)	0.45
Atrial fibrillation	150 (0.50)	3 (0.34)	0.81
Chronic hepatitis	2900 (9.65)	78 (8.72)	0.38
COPD	1365 (4.54)	42 (4.70)	0.89
Hypoglycemia	118 (0.39)	7 (0.78)	0.10
Albuminuria	297 (0.99)	6 (0.67)	0.44
Retinopathy	583 (1.94)	31 (3.47)	0.002
Cardiovascular risk factors, n (%)			
SBP ≥ 130/DBP ≥85 (mmHg)	20,730 (69.01)	607 (67.90)	0.50
TG ≥ 150 (mg/dL)	13,075 (43.53)	397 (44.41)	0.63
HDL: female < 50; male < 40 (mg/dL)	15,595 (51.92)	448 (50.11)	0.30
LDL ≥ 100 (mg/dL)	20,929 (69.68)	618 (69.13)	0.75
eGFR < 60 (mL/min/1.73 m^2^)	7967 (26.52)	240 (26.85)	0.86

Differences in continuous variables were tested using the student’s *t*-test. Differences in categorical variables were tested using the Chi-square test or Fisher’s exact test

*CHF* congestive heart failure, *CAD* coronary artery disease, *SBP* systolic blood pressure, *DBP* diastolic blood pressure, *COPD* chronic obstructive pulmonary disease, *HDL* high-density lipoprotein, *TG* triglyceride, *eGFR* estimated glomerular filtration rate, *LDL* low-density lipoprotein

**Fig. 1:**
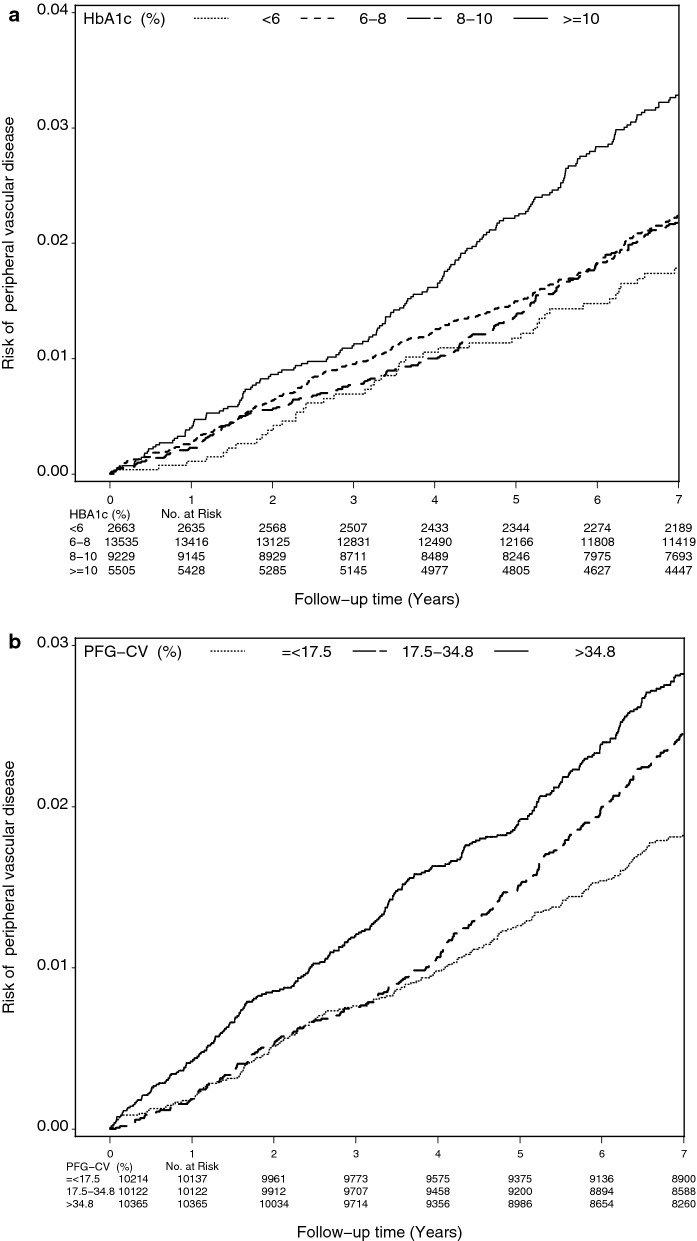
Risk of peripheral vascular disease by **a** HbA1c level and **b** FPG-CV level

Table 2 shows baseline characteristics in participants grouped according to tertiles of FPG-CV. Persons with lower FPG-CV were found to be associated with higher proportion of male and obesity, lower mean diabetes duration and HbA1c, and lower prevalence of smoking, 3 or more oral hypoglycemic drug use, insulin injections, insulin injections plus oral hypoglycemic drug use, stroke, cancer, hypertension, COPD, and hypoglycemia, as well as higher prevalence of 1 oral hypoglycemic drug use, 2 oral hypoglycemic drug use, and hyperlipidemia. Figure 1 depicts the cumulative risk for PAD estimated by Kaplan–Meier method within subgroups defined by HbA1c and FPG-CV; patients with HbA1c ≥ 10% or FPG-CV > 34.8% had an increased risk of PAD (both log-rank tests p < 0.001).

**Table 2:** Baseline characteristics based on HbA1c level and FPG-CV in patients with type 2 diabetes enrolled in the National Diabetes Care Management Program, Taiwan (n = 30,932)

Variables	HbA1c (%)					FPG-CV (%)			*p*-value
	< 6.0 (N = 2663)	6.0–8.0 (N = 13,535)	8.0–10.0 (N = 9229)	≥ 10.0 (N = 5505)	*p*-value	≤ 17.5 (N = 10,214)	17.5–34.8 (N = 10,199)	> 34.8 (N = 10,519)	
Sociodemographic factors									
Male, n (%)	1496 (56.18)	6406 (47.33)	3959 (42.9)	2568 (46.65)	< 0.001	4880 (47.78)	4645 (45.54)	4904 (46.62)	0.006
Age (years), mean (SD)	62.84 (11.93)	62.11 (11.02)	60.52 (10.85)	58.27 (11.03)	< 0.001	61.07 (11.13)	61.04 (11.03)	60.93 (11.29)	0.64
Lifestyle behaviors, n (%)									
Smoking	377 (14.16)	1897 (14.02)	1394 (15.1)	992 (18.02)	< 0.001	1407 (13.78)	1479 (14.50)	1774 (16.86)	< 0.001
Alcohol drinking	255 (9.58)	1133 (8.37)	730 (7.91)	467 (8.48)	0.05	858 (8.4)	818 (8.02)	909 (8.64)	0.27
Diabetes-related variables									
Duration of diabetes (years), mean (SD)	5.00 (5.98)	6.60 (6.70)	7.69 (7.01)	6.84 (6.69)	< 0.001	6.13 (6.32)	7.01 (6.78)	7.35 (7.13)	< 0.001
Type of hypoglycemic drug use, n (%)					< 0.001				< 0.001
No medication	104 (3.91)	180 (1.33)	57 (0.62)	29 (0.53)		177 (1.73)	101 (0.99)	92 (0.87)	
One oral hypoglycemic drug	991 (37.21)	2993 (22.11)	866 (9.38)	397 (7.21)		2473 (24.21)	1566 (15.35)	1208 (11.48)	
Two oral hypoglycemic drugs	1135 (42.62)	6412 (47.37)	3640 (39.44)	1702 (30.92)		4520 (44.25)	4441 (43.54)	3928 (37.34)	
Three oral hypoglycemic drugs	234 (8.79)	2086 (15.41)	2114 (22.91)	1278 (23.22)		1663 (16.28)	2044 (20.04)	2005 (19.06)	
> 3 oral hypoglycemic drugs	40 (1.50)	485 (3.58)	635 (6.88)	474 (8.61)		423 (4.14)	587 (5.76)	624 (5.93)	
Insulin	55 (2.07)	327 (2.42)	332 (3.6)	189 (3.43)		175 (1.71)	237 (2.32)	491 (4.67)	
Insulin+ oral hypoglycemic drug	104 (3.91)	1052 (7.77)	1585 (17.17)	1436 (26.09)		783 (7.67)	1223 (11.99)	2171 (20.64)	
HbA1c (%), mean (SD)	5.47 (0.45)	6.99 (0.55)	8.83 (0.57)	11.43 (1.28)	< 0.001	7.69 (1.73)	8.12 (1.80)	8.76 (2.13)	< 0.001
FPG-CV (%),mean (SD)	26.29 (23.24)	27.83 (23.21)	33.92 (25.26)	43.14 (31.76)	< 0.001	10.15 (4.72)	25.41 (4.90)	60.30 (25.79)	< 0.001
Drug-related variables, n (%)									
Hypertension drug treatment	1063 (39.92)	5572 (41.17)	3610 (39.12)	1742 (31.64)	< 0.001	4005 (39.21)	3960 (38.83)	4022 (38.24)	0.35
Comorbidity, n (%)									
Obesity (BMI ≥ 27)	961 (36.09)	5066 (37.43)	3439 (37.26)	1765 (32.06)	< 0.001	3796 (37.16)	3756 (36.83)	3679 (34.97)	0.002
CAD	226 (8.49)	1241 (9.17)	839 (9.09)	396 (7.19)	< 0.001	884 (8.65)	906 (8.88)	912 (8.67)	0.81
Stroke	169 (6.35)	654 (4.83)	497 (5.39)	251 (4.56)	0.002	432 (4.23)	516 (5.06)	623 (5.92)	< 0.001
CHF	83 (3.12)	375 (2.77)	227 (2.46)	119 (2.16)	0.03	240 (2.35)	280 (2.75)	284 (2.70)	0.15
Cancer	56 (2.10)	280 (2.07)	216 (2.34)	97 (1.76)	0.13	191 (1.87)	206 (2.02)	252 (2.40)	0.02
Hyperlipidemia	627 (23.54)	3592 (26.54)	2414 (26.16)	1208 (21.94)	< 0.001	2738 (26.81)	2619 (25.68)	2484 (23.61)	< 0.001
Hypertension	1282 (48.14)	6565 (48.50)	4296 (46.55)	1990 (36.15)	< 0.001	4545 (44.5)	4769 (46.76)	4819 (45.81)	0.005
Atrial fibrillation	18 (0.68)	66 (0.49)	46 (0.50)	23 (0.42)	0.48	47 (0.46)	46 (0.45)	60 (0.57)	0.39
Chronic hepatitis	268 (10.06)	1332 (9.84)	899 (9.74)	479 (8.70)	0.08	941 (9.21)	992 (9.73)	1045 (9.93)	0.19
COPD	138 (5.18)	621 (4.59)	427 (4.63)	221 (4.01)	0.10	397 (3.89)	468 (4.59)	542 (5.15)	< 0.001
Hypoglycemia	17 (0.64)	65 (0.48)	36 (0.39)	7 (0.13)	0.001	24 (0.23)	39 (0.38)	62 (0.59)	< 0.001
Cardiovascular risk factors, n (%)									
SBP ≥ 130/DBP ≥ 85 (mmHg)	1811 (68.01)	9480 (70.04)	6459 (69.99)	3587 (65.16)	< 0.001	7056 (69.08)	7044 (69.07)	7237 (68.80)	0.88
TG ≥ 150 (mg/dL)	934 (35.07)	5460 (40.34)	4319 (46.8)	2759 (50.12)	< 0.001	4166 (40.79)	4390 (43.04)	4916 (46.73)	< 0.001
HDL: female < 50; male < 40 (mg/dL)	1315 (49.38)	7036 (51.98)	4955 (53.69)	2737 (49.72)	< 0.001	5178 (50.7)	5358 (52.53)	5507 (52.35)	0.01
LDL ≥ 100 (mg/dL)	1688 (63.39)	9228 (68.18)	6521 (70.66)	4110 (74.66)	< 0.001	7202 (70.51)	7056 (69.18)	7289 (69.29)	0.07
eGFR < 60 (mL/min/1.73 m^2^)	827 (31.06)	3858 (28.5)	2333 (25.28)	1189 (21.6)	< 0.001	2340 (22.91)	2661 (26.09)	3206 (30.48)	< 0.001

Differences in continuous variables tested by ANOVA. Differences in categorical variables tested by chi-square or Fisher’s exact test

*CHF* congestive heart failure, *CAD* coronary artery disease, *SBP* systolic blood pressure, *DBP* diastolic blood pressure, *COPD* chronic obstructive pulmonary disease, *HDL* high-density lipoprotein, *TG* triglyceride, *eGFR* estimated glomerular filtration rate, *LDL* low-density lipoprotein

**Fig. 2:**
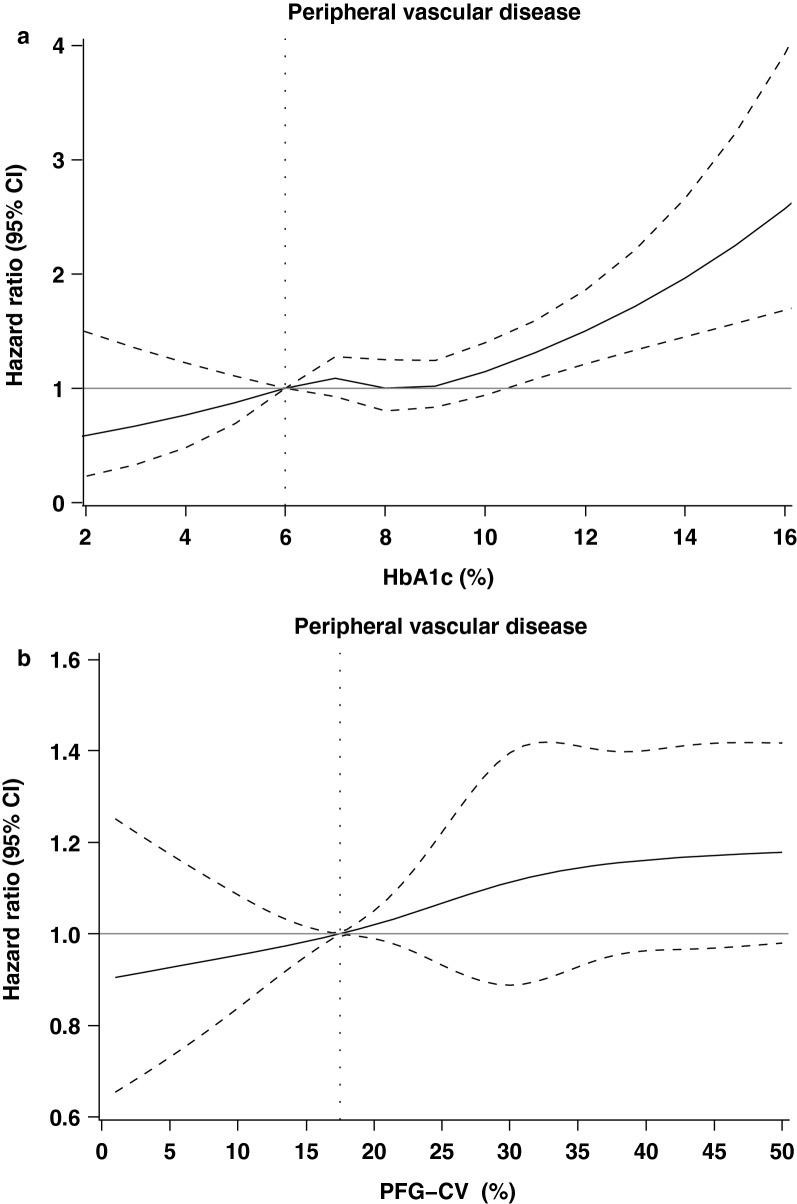
Restricted multivariable cubic spline plots for **a** HbA1c level and **b** FPG-CV level (n = 30,932). HRs were adjusted for age, sex, alcohol consumption, smoking, diabetes duration, type of hypoglycemic medication, antihypertensive medication, obesity, cardiovascular risk factors, coronary artery disease, stroke, cancer, congestive heart failure, hyperlipidemia, atrial fibrillation, hypertension, chronic obstructive pulmonary disease, chronic hepatitis, and hypoglycemia

Table 3 shows the HRs of PAD based on subgroups of FPG-CV, with and without adjustment for lifestyles, FPG, and comorbidity. FPG-CV was associated with PAD risks. We observed an independent association between FPG-CV in the third tertile and PAD incidence in the third multivariate model, with an HR of 1.24 (95% CI 1.04–1.47), and found that HbA1c ≥ 10% was also independently associated with increased PAD risk (HR: 1.50, 95% CI 1.00–2.40, p < 0.05). With using restricted cubic splines, we observed a non-linear increasing association between HbA1c and risk of PAD, with leveling off at 6-9%; a linear increasing trend for FPG-CV was also observed, with leveling off at ≥ 35% (Fig. 2). Based on the statistics of likelihood ratio tests for HbA1c and FPG-CV, we found HbA1c had a higher predictive power on PAD than FPG-CV (12.51 vs. 6.0) whereas FPG-CV had a higher predictive power on death (54.62 vs 112.69).

**Table 3:** Hazard ratios (HRs) of peripheral vascular disease based on HbA_1_c and FPG-CV levels in persons with diabetes enrolled in the National Diabetes Care Management Program, Taiwan (n = 30,932)

Variables	n	Cases	Person-years	IR	Peripheral vascular disease (N = 894)		
					Age and gender-adjusted	Multivariate-adjusted^a^	Multivariate-adjusted^b^
HbA1c (%)							
< 6	2663	59	21,708.26	2.72	1.00	1.00	1.00
6–8	13,535	377	111,972.54	3.37	2.31 (1.72, 3.11)***	1.20 (0.90, 1.58)	1.19 (0.90, 1.57)
8–10	9229	247	75,713.95	3.26	2.29 (1.69, 3.10)***	1.06 (0.79, 1.42)	1.05 (0.78, 1.41)
≥ 10	5505	211	44,117.07	4.78	3.52 (2.59, 4.76)***	1.52 (1.12, 2.06)**	1.50 (1.10, 2.04)**
p for trend					< 0.001	0.01	0.01
FPG-CV (%)							
≤ 17.5	10,214	251	85,777.39	2.93	1.00	1.00	1.00
17.5–34.8	10,199	306	84,671.99	3.61	1.34 (1.13, 1.59)***	1.18 (1.00, 1.40)	1.17 (0.99, 1.39)
> 34.8	10,519	337	83,062.43	4.06	1.44 (1.21, 1.71)***	1.25 (1.05, 1.48)*	1.24 (1.04, 1.47)*
p for trend					< 0.001	0.01	0.01

Multivariate-adjusted^a^ for age, gender, alcohol consumption, smoking, diabetes duration, type of hypoglycemic medication, hypertension medication and obesity

Multivariate-adjusted^b^ for cardiovascular risk factors, stroke, coronary artery disease, cancer, congestive heart failure, hyperlipidemia, atrial fibrillation, hypertension, chronic hepatitis, hypoglycemia and chronic obstructive pulmonary disease in addition to the variables in first multivariate model

*IR* incidence density rate = number of incident cases/person-years * 1000

* p < 0.05; ** p < 0.01; *** p < 0.001

Sensitivity analysis demonstrated that the PAD HRs remained similar for patients in the second to third tertiles of FPG-CV [1.01 (95% CI 1.02–1.44) and 1.27 (95% CI 1.07–1.52)]. The linear trend for the risk of PAD remained significant for FPG-CV, even after patients with various types of comorbidity were excluded (p for trend = 0.006) (Table 4).

**Table 4:** Sensitivity analyses for evaluating the potential bias due to comorbidities, by excluding persons with diabetic ketoacidosis, hyperglycemic hyperosmolar nonketotic coma, atrial fibrillation, myocardial infarction, and hypoglycemia

Variables	n	Cases	Person-years	IR	Multivariate-adjusted HR 95% CI
Model I					
HbA1c (%)					
< 6	2607	56	21,284.98	2.63	1.00
6–8	13,335	368	110,444.51	3.33	1.21 (0.91, 1.61)
8–10	9077	242	74,554.23	3.25	1.07 (0.79, 1.45)
≥ 10	5386	205	43,260.94	4.74	1.53 (1.11, 2.09)**
p for trend					0.01
FPG-CV (%)					
≤ 17.5	10,074	244	84,668.74	2.88	1.00
17.5–34.8	10,048	303	83,481.24	3.63	1.19 (1.01, 1.42)*
> 34.8	10,283	324	81,394.68	3.98	1.23 (1.04, 1.47)*
p for trend					0.02
Model II					
HbA1c (%)					
< 6	2629	58	21,421.83	2.71	1.00
6–8	13,412	372	110,998.85	3.35	1.18 (0.89, 1.56)
8–10	9144	245	75,038.39	3.26	1.04 (0.77, 1.40)
≥ 10	5436	211	43,554.03	4.84	1.51 (1.11, 2.05)**
p for trend					0.01
FPG-CV (%)					
≤ 17.5	10,126	246	85,067.16	2.89	1.00
17.5–34.8	10,105	304	83,885.01	3.62	1.19 (1.00, 1.41)*
> 34.8	10,390	336	82,060.93	4.09	1.26 (1.06, 1.50)**
p for trend					0.007
Model III					
HbA1c (%)					
< 6	2604	59	21,261.14	2.78	1.00
6–8	13,181	372	109,131.66	3.41	1.18 (0.89, 1.57)
8–10	8981	245	73,772.11	3.32	1.05 (0.78, 1.41)
≥ 10	5365	204	43,072.94	4.74	1.47 (1.08, 2.00)*
p for trend					0.02
FPG-CV (%)					
≤ 17.5	9968	248	83,751.3	2.96	1.00
17.5–34.8	9938	301	82,597.88	3.64	1.17 (0.99, 1.39)
> 34.8	10,225	331	80,888.67	4.09	1.24 (1.04, 1.47)*
p for trend					0.01
Model IV					
HbA1c (%)					
< 6	2645	59	21,577.48	2.73	1.00
6–8	13,469	374	111,541.73	3.35	1.18 (0.89, 1.56)
8–10	9183	247	75,373.75	3.28	1.05 (0.78, 1.41)
≥ 10	5482	211	43,970.42	4.80	1.50 (1.11, 2.04)**
p for trend					0.01
FPG-CV (%)					
≤ 17.5	10,167	249	85,419.13	2.92	1.00
17.5–34.8	10,153	305	84,359.17	3.62	1.18 (0.99, 1.39)
> 34.8	10,459	337	82,685.08	4.08	1.25 (1.05, 1.48)*
p for trend					0.009
Model V					
HbA1c (%)					
< 6	2646	59	21,595.5	2.73	1.00
6–8	13,470	372	111,547.16	3.33	1.17 (0.88, 1.54)
8–10	9193	246	75,477.85	3.26	1.03 (0.77, 1.39)
≥ 10	5498	210	44,089.87	4.76	1.46 (1.08, 1.99)*
p for trend					0.02
FPG-CV (%)					
≤ 17.5	10,190	248	85,621.37	2.90	1.00
17.5–34.8	10,160	304	84,395.44	3.60	1.18 (1.00, 1.40)
> 34.8	10,457	335	82,693.58	4.05	1.25 (1.05, 1.48)*
p for trend					0.008
Model VI					
HbA1c (%)					
< 6	2500	56	20,470.2	2.74	1.00
6–8	12,803	352	106,329.25	3.31	1.15 (0.86, 1.54)
8–10	8716	239	71,749.68	3.33	1.06 (0.78, 1.43)
≥ 10	5185	197	41,763.28	4.72	1.46 (1.06, 2.00)*
p for trend					0.02
FPG-CV (%)					
≤ 17.5	9734	235	81,922.2	2.87	1.00
17.5–34.8	9657	293	80,391.77	3.64	1.21 (1.02, 1.44)*
> 34.8	9813	316	77,998.43	4.05	1.27 (1.07, 1.52)**
p for trend					0.006

Multivariate-adjusted for age, gender, alcohol consumption, smoking, diabetes duration, type of hypoglycemic medication, hypertension medication, obesity cardiovascular risk factors, stroke, coronary artery disease, cancer, congestive heart failure, hyperlipidemia, atrial fibrillation, hypertension, chronic hepatitis, hypoglycemia and chronic obstructive pulmonary disease

Model I: persons with HHNK being excluded (N = 527)

Model II: persons with DKA being excluded (N = 311)

Model III: persons with myocardial infarction being excluded (N = 801)

Model IV: persons with atrial fibrillation being excluded (N = 153)

Model V: persons with hypoglycemia being excluded (N = 125)

Model VI: persons with HHNK, DKA, myocardial infarction, atrial fibrillation and hypoglycemia being excluded (N = 1728)

* p < 0.05; ** p < 0.01; *** p < 0.001

## Discussion

This study investigated HbA1c levels and FPG variability in relation to PAD in persons with type 2 diabetes. We identified a non-conventional risk factor, FPG-CV, was associated with greater risk for PAD in persons with type 2 diabetes during a follow-up period of 8.2 years. Moreover, our findings demonstrated an association between FPG-CV and PAD incidence, independent of HbA1c. These findings remained consistent in sensitivity analysis by excluding potential confounders, demonstrating the robustness of our study results.

Our study’s strengths include use of a large national population database of type 2 diabetes cases, a retrospective cohort study design with a relatively long follow-up period, standard data collection procedures, and adjusted for a large numbers of candidate confounders, which further validate our results. Our findings are crucial to the clinical management of PAD in type 2 diabetes. First, glycemic variability as determined by FPG-CV levels may be considered an additional non-conventional risk factor for PAD in type 2 diabetes. Second, the current practice of relying mainly on HbA1c alone but not glycemic variability may be inadequate. It may be necessary to achieve both a glycemic target and glycemic control stability. Further well-designed studies are warranted to determine whether minimizing glycemic variability has a crucial impact on PAD development in type 2 diabetes.

PAD increases diabetes-related complications; therefore, recognizing the risk factors for PAD is crucial [4, 23]. Prior studies’ evidence indicates elevated HbA1c is an independent determinant for PAD in type 2 DM. The present study confirms previous findings that elevated HbA1c is an independent factor for PAD in persons with type 2 diabetes [1, 3, 23, 24]. Nonetheless, studies on the benefit of lowering blood glucose alone, using HbA1c as a therapeutic target, did not show improvement in claudication symptoms or slowing of PAD progression in type 2 diabetes [25]. Interpretation of this finding is complicated, partly because HbA1c level merely presents the average blood glucose in the preceding 8–12 weeks and fails to reflect variability in glycemia, which may indicate an additional factor for development of diabetic vascular complications independent of HbA1c [6, 26]. Our results add to the emerging concept that glycemic variability as determined by FPG-CV confers additional risk of PAD, indicating the need for further research.

Numerous processes are linked to the development of PAD, which is considered a marker of systemic atherosclerosis [1, 3, 23, 24]. Conventional atherosclerotic risk factors, consisting of age, diabetes, smoking, hypertension, dyslipidemia, microalbuminuria, and obesity are associated with PAD [3, 4, 23, 27]. Several potential mechanisms may link glycemic variability to the occurrence of PAD from a pathophysiological point of view. First, evidence has suggested that oscillating glucose may trigger increases in oxidative stress, endothelial dysfunction or damage, advanced glycation end-products and inflammatory cytokines, resulting in a higher rate of occurrence and acceleration of atherosclerosis independently of sustained hyperglycemia [12–14, 28–33]. Second, A relationship of cardiovascular autonomic dysfunction with atherosclerosis has been observed [34, 35]. Exaggerated glycemic fluctuations were demonstrated to adversely affect endothelial vessel hearing with result of hypoxia and blood flow disorders in neuronal cells, which may lead to autonomic dysfunction [36]. Previous study has revealed that patients with type 2 diabetes with PAD had lower heart rate variability (HRV) indices than patients without PAD, which reflects a cardiovascular autonomic dysfunction [34]. Moreover, visit to visit variability in FPG could be an important risk factor for long term changes in left cardiac structure and function in patients with type 2 DM [37]. In this regard, the associations between PAD and cardiovascular autonomic dysfunction could be partly explained by glycemic fluctuations. Third, glycemic fluctuations might cause insulin resistance, beta cell dysfunction and apoptosis, inducing cellular metabolic memory, and epigenetic changes [12–14, 38]. According to these findings, we hypothesize that variability in glycemia may be a crucial pathological mechanism in PAD. Future research is warranted to explore the plausible biological mechanism by which glycemic variability contributes to the pathogenesis of PAD.

The current study has several limitations. First, given its observational nature, the possibility of selection bias and unrecognized confounding variables may have affected the findings, despite use of multivariate regression analysis. Second, glycemic variability can be assessed using a variety of methods, and FPG-CV is only one such method. To date, there is little consensus about a preferred method for assessment of the role of glycemic variability in diabetic complications. Methods for precise measurement of glycemic variability are also needed to compare the predictive ability of each method for diabetic complications including PAD [6, 39]. Third, we only considered the 1st-year FPG-CV and could not consider time-varying FPG-CV because the data was not availability. FPG-CV may have been changeable, while the doctors are treating. Thus, we examined the concordance of DM treatment between the 1st year and the 2nd year after entry to the study and found all types of anti-diabetes medications use had agreement greater than 90%, ranging from 94 to 99%. Fourth, the diagnosis of PAD was not validated through reviewing medical records and was only determined by ICD codes, and thus relied on the accuracy of diagnostic in our database. The NHI Bureau in Taiwan selects a sample of medical records routinely and endeavors to verify the accuracy of diagnoses in the database, thereby improving the accuracy of coding [17, 18]. The insurance claims are routinely scrutinized by medical specialists under anonymous peer review based on standard diagnostic criteria, and coding errors are penalized. Further, to improve accurate diagnosis of PAD, we included only those incident cases in which medical service was administered for PAD during either ≥ 3 outpatient visits or ≥ 1 hospitalization. Similar methods for PAD ascertainment have been adopted by previous studies [40, 41]. Fifth, PAD is often asymptomatic [42], and asymptomatic PAD or mild PAD symptoms might have been overlooked. Therefore, the risk of PAD could be underestimated. In this study, we only considered patients with symptoms and those seeking medical care, i.e., those diagnosed in clinical practice. Fifth, the effects of glycemic variability on PAD severity were not examined. Further studies are needed to determine whether the effects of glycemic variability are correlated with ABI severity. Finally, all of our study subjects in our study were ethnic Chinese from Taiwan, and generalizability of our results to other ethnic groups needs to be further confirmed.

## Conclusions

Both visit-to-visit variability in glycemia, determined by FPG-CV level, and HbA1c represented risk factors for PAD besides other conventional risk factors in persons with type 2 diabetes. Our study’s findings show that HbA1c level and glucose fluctuation may be targeted for PAD prevention in type 2 diabetes. Further study is required to clarify the underlying mechanisms and verify whether FPG-CV or HbA1c level is a valuable therapeutic target.

## Supplementary information

**Additional file 1: Figure S1.** Flow chart of recruitment procedures for the study. **Table S1.** Hazard ratios (HRs) of peripheral vascular disease based on HbA_1_c and FPG-CV levels in persons with diabetes enrolled in the National Diabetes Care Management Program, Taiwan (n=30,932).

## Data Availability

The datasets generated and/or analyzed during the current study are not publicly available due to the policy declared by National Health Insurance in Taiwan but are available from the corresponding author on reasonable request.
