# Supplementary material for: Fasting plasma glucose variability and HbA1c are associated with peripheral artery disease risk in type 2 diabetes
Source: Cardiovasc Diabetol. 2020 Jan 7;19:4. doi: 10.1186/s12933-019-0978-y (PMC6945758; doi:10.1186/s12933-019-0978-y)
Supplement: Supplementary file 1 — Additional file 1: Figure S1. Flow chart of recruitment procedures for the study. Table S1. Hazard ratios (HRs) of peripheral vascular disease based on HbA1c and FPG-CV levels in persons with diabetes enrolled in the National Diabetes Care Management Program, Taiwan (n=30,932). [file 12933_2019_978_MOESM1_ESM.pdf]

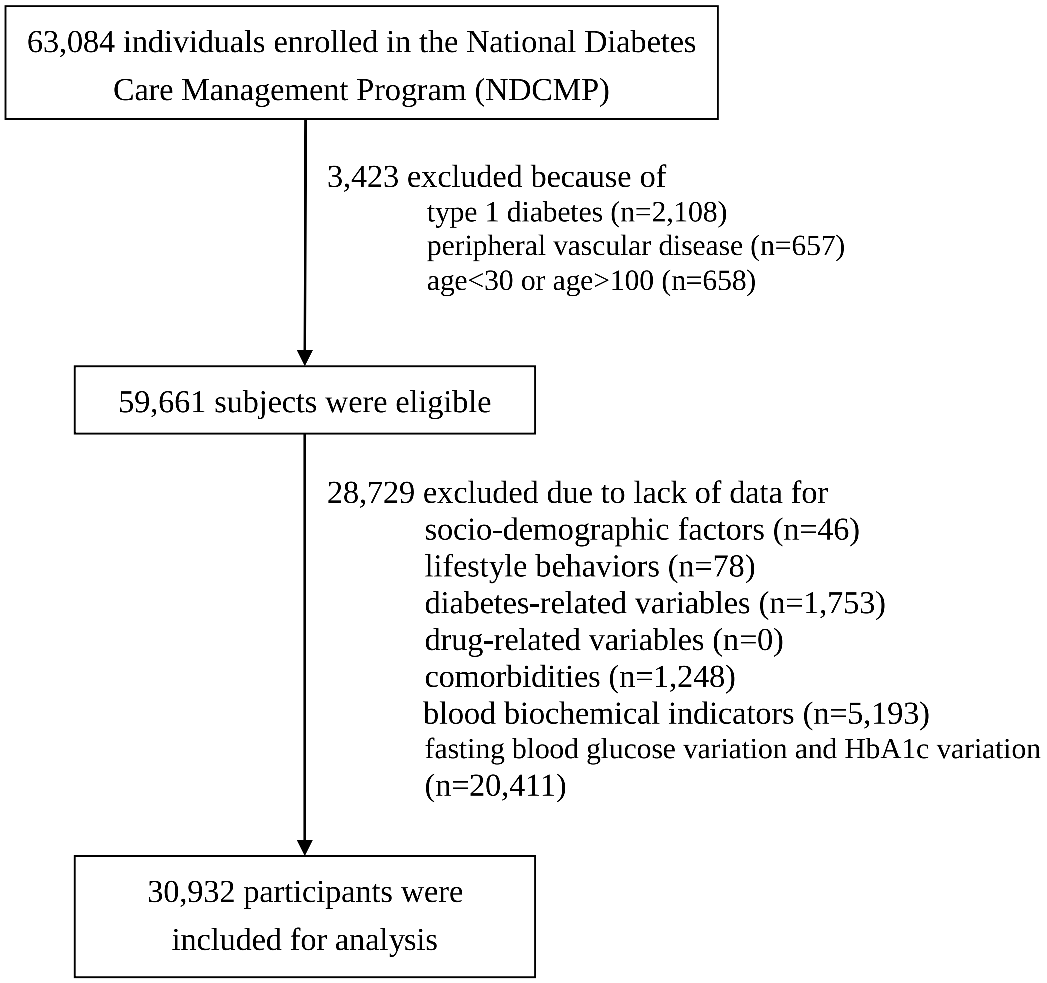

Figure S1. Flow chart of recruitment procedures for the study

Table S1. Hazard ratios (HRs) of peripheral vascular disease based on HbA_1c and FPG-CV levels in persons with diabetes enrolled in the National Diabetes Care Management Program, Taiwan (n=30,932)

	Peripheral vascular disease (N=894)			
Variables	Multivariate-Adjusted[1]	Multivariate-Adjusted[2]	Multivariate-Adjusted[1]	Multivariate-Adjusted[2]
HbA1c (%)	**Baseline HbA1c**		**Mean first-year HbA1c**	
<6	1.00	1.00	1.00	1.00
6~8	1.20 (0.90, 1.58)	1.19 (0.90, 1.57)	0.98 (0.73, 1.32)	0.96 (0.72, 1.29)
8~10	1.06 (0.79, 1.42)	1.05 (0.78, 1.41)	1.12 (0.83, 1.52)	1.08 (0.79, 1.48)
≥10	1.52 (1.12, 2.06)**	1.50 (1.10, 2.04)**	1.38 (0.98, 1.94)	1.29 (0.90, 1.84)
P for trend	0.01	0.01	0.002	0.02
FPG-CV (%)				
≤ 17.5	1.00	1.00	1.00	1.00
17.5-34.8	1.18 (1.00, 1.40)	1.17 (0.99, 1.39)	1.17 (0.99, 1.38)	1.16 (0.98, 1.38)
>34.8	1.25 (1.05, 1.48)*	1.24 (1.04, 1.47)*	1.24 (1.05, 1.47)*	1.23 (1.03, 1.45)*
P for trend	0.01	0.01	0.009	0.02

*:p<0.05; **:P<0.01; ***:p<0.001.

Multivariate-adjusted[1] for age, gender, alcohol consumption, smoking, diabetes duration, type of hypoglycemic medication, hypertension medication and obesity.

Multivariate-adjusted[2] for cardiovascular risk factors, stroke, coronary artery disease, cancer, congestive heart failure, hyperlipidemia, atrial fibrillation, hypertension, chronic hepatitis, hypoglycemia, chronic obstructive pulmonary disease and baseline fasting plasma glucose in addition to the variables in first multivariate model.

IR: incidence density rate = number of incident cases/person-years*1000.
